# Work-related injuries in Qatar for 1 year: an initial report from the work-related injury unified registry for Qatar 

**DOI:** 10.5339/qmj.2022.58

**Published:** 2022-12-05

**Authors:** Rafael Consunji, Ayman El-Menyar, Nazia Hirani, Aisha Abeid, Hassan Al-Thani, Muhammad S Hardan, Sailesh Chauhan, Hasan Kasem, Ruben Peralta

**Affiliations:** ^1^Hamad Injury Prevention Program, Hamad Trauma Centre, Department of Surgery, Hamad General Hospital, Hamad Medical Corporation, Doha, Qatar. Email and ORCID ID: rconsunji@hamad.qa & https://orcid.org/0000-0002-9854-3585; ^2^Clinical Research, Trauma & Vascular Surgery, Hamad General Hospital, Doha, Qatar Clinical Medicine, Weill Cornell Medical College, Doha, Qatar.; ^3^Trauma and Vascular Surgery Section, Department of Surgery, Hamad General Hospital, Hamad Medical Corporation, Doha, Qatar.; ^4^Ambulance Services, Hamad Medical Corporation, Doha, Qatar.; ^5^Qatar Red Crescent, Doha, Qatar.; ^6^Department of Surgery, Universidad Nacional Pedro Henriquez Urena, Santo Domingo, Dominican Republic.

**Keywords:** work-related injuries, registry, worker safety

## Abstract

Background: The Ministry of Public Health National Health Strategy 2018–2022 has recognized the need for accurate, updated, and representative data that truly reflects the occupational health and safety status in Qatar. In 2015, the Hamad Trauma Center received a research grant to create a unified registry for work-related injuries in Qatar [WURQ], whose processes and research findings have been reported earlier. This paper shall describe the findings from the initial 1-year collection of data on work-related injuries [WRIs] and deaths in Qatar for the year 2020 through the WURQ database.

Methods: The WURQ database was queried for all WRIs from January 1 to December 31, 2020. These data were classified by date of injury, age, sex, nationality, mechanism of injury, severity of injury, location of medical consultation, and clinical outcome.

Results: Out of a total worker population of 2,174,828 [2.29 occupational fatalities per 100,000 workers, there were 50 deaths caused by WRIs]. The majority of WRI deaths were in the prehospital setting [60%] with the majority of fatal injuries occurring at the worksite [64%] and 22% due to falls. Five hundred six workers sustained severe WRIs [23.26 severe occupational injuries per 100,000 workers], and 37,601 workers sustained mild to moderate WRIs [1,728.91 mild to moderate occupational injuries per 100,000 workers]. The severe WRIs were most commonly due to falls [226 out of 506] from height [45%] and falling heavy objects [80 out of 506] [16%]. Road traffic injuries [RTI] make up one-fourth [133 out of 506] of all severe WRIs.

Conclusion: WURQ has described WRIs in Qatar using a purpose-built and nationally linked occupational injury registry. Occupational injury and injury fatality statistics, for Qatar in 2020, are lower than or comparable with those from other high-income countries. This data can be used to inform worksite inspections, investigations, worker safety education, environmental improvements, and injury prevention programs to make Qatar safer for all its workers.

## Introduction

The status of occupational health and safety [OHS] in Qatar has drawn attention and scrutiny from the popular media, more so in the light of the upcoming FIFA World Cup that will be held there in November and December 2022.^1^ Many of these reports are designed as clickbait and based on questionable and shoddy data.

Common approaches include cherry-picking sensational cases, using nonstandard definitions of work-related death or injury, using nonrepresentative and incomplete data sources, and making sensational sweeping generalizations based on any or all of the aforementioned.^2,3^ Regardless of which methodologies these reports used, clearly none of them would meet the strict criteria of a peer-reviewed academic publication, and there is a great need for an objective and empiric report that more accurately describes the status of OHS in Qatar.

In contrast to the above approaches, the use of globally compliant definitions, indicators, and data sources will allow the temporal and international comparison of WRI statistics and provide real-world data for reporting. The use of incomplete and nonrepresentative WRI data results in unreliable and inaccurate reports. This study will focus on this “accuracy” gap.

The need for accurate, updated, and representative data that truly reflects the OHS status in Qatar has long been recognized as a public health and research priority in the Ministry of Public Health [MoPH] National Health Strategy 2018–2022.^3^ According to state policy and labor law, all companies in Qatar are required to report any occupational injury or accident at work.^5,6^ While the Ministry of Labor [MOL] collects this data for inspection and policymaking purposes, it is acknowledged that compliance with these laws is an area in need of improvement.

As a result, the Hamad Trauma Center [HTC] of the Hamad Medical Corporation [HMC] was awarded with a research grant in 2015 [#7-1120-3–288 “A Unified Registry for Occupational Injury Prevention in Qatar”] from the Qatar Foundation National Priorities Research Program. This grant was specifically designed to create a WRI Unified Registry for Qatar [WURQ] to serve as a viable and sustainable database to provide representative, updated, and accurate data on work-related injuries [WRIs] and deaths. The processes and research findings of this grant have been reported previously.^7-9^

Subsequently, the original NPRP grant was extended for an initial 3-month period [February–April 2020, WURQ2] by the Medical Research Center of HMC, then for an additional 6-month period, June–November 2020 and a final 3-month period, January, May, and December 2020 [WURQ3], supported by a grant from the Qatar Office of the International Labour Organization [ILO] [Supplementary file: Appendix A].

The overall objective of WURQ2 was to establish “proof of concept” that the WURQ is a viable and sustainable database that can provide an estimate on workload, staffing, and administrative needs for the same, for future planning and possible institutionalization or adoption by an appropriate government ministry. The overall objective of WURQ3 was to use the WURQ data as the basis for both strategic and specific interventions by the OHS Unit of the Labour Inspection Department in the MOL, formerly known as the Ministry of Administrative Development, Labor, and Social Affairs [Supplementary file: Appendix B and Appendix C].

Previous publications provide the framework, lessons learned, and direct healthcare costs of WRIs in Qatar.^7-9^ This paper shall add to this information by describing the findings from the initial 1-year collection, through the WURQ registry, of data on work-related injuries and deaths in Qatar for the year 2020.

## Methods

The HMC is the main provider of secondary and tertiary healthcare in Qatar and the only provider of acute and emergency care for victims of traumatic injury. The HTC sees and treats almost 3000 patients with moderate to severe traumatic injury every year, representing at least 98% of all trauma victims in Qatar. The emergency departments [EDs] of HMC treat almost 25,000 patients with various urgent care emergencies on a daily basis, with a varying proportion of them with traumatic injuries. The Qatar Red Crescent [QRC] clinics are located in the industrial area, closer to the areas of construction and industry in Qatar, and they see more than 3000–4000 patients with mild to moderate injuries every day. Almost 100% of their patients are craft and manual workers.

The WURQ grant implemented a mandatory data linkage tool, which identified all patients with WRIs who presented acutely, in the electronic health record system of five HMC hospital EDs the HTC trauma registry, the HMC Ambulance Service [HMCAS], and the QRC, as previously described.^8^ Patients with WRIs are “tagged” using a mandatory tool that was filled in using a standard WRI definition by an ED nurse, emergency medical services prehospital provider, or QRC clinic nurse. The mandatory nature of this identification tool was essential so that no patient, with a WRI, could be discharged, admitted, or transferred from their point of initial consultation for acute care without this data field being completely filled in.

The following are the definitions of WRI applied in the WURQ [source, ILO]^10^

• An occupational injury is defined as any personal injury, disease, or death resulting from an occupational accident.
• An occupational accident is an unexpected and unplanned occurrence, including acts of violence and arising out of or in connection with work, which results in one or more workers incurring a personal injury, disease, or death.
• A case of occupational injury is the case of one worker incurring an occupational injury because of one occupational accident. An occupational injury could be fatal [because of occupational accidents and where death occurred within 1 year of the day of the accident] or nonfatal with lost work time.

The WURQ database was queried for all WRIs seen and treated or admitted at any HMC ED, the HTC, or QRC clinic, from January 1 to December 31, 2020. The HMCAS provided data on all WRIs they saw, treated, or transported for the same study period.

Data encoded and the sources utilized were as follows:

a. Moderate to severe WRIs, defined as “any WRI needing treatment at, admission to, or resulting in an in-hospital death at the HTC.”^11^
b. Mild to moderate WRIs, defined as “any WRI needing a clinic visit or urgent consultation at an emergency room,”^11^ were collected from the five HMC EDs [Hamad General Hospital, Al Wakra Hospital, The Cuban Hospital, Al Khor Hospital, or Hazm Mebaireek General Hospital] or four QRC clinics [Al Hemaila, Mesaimeer, Zekreet, and Fereej Abdel Aziz Health Centers]. The QRC clinics only see and treat male workers, officially referred to as craft manual workers, who work in blue-collar jobs and industries.
c. WRIs transported or classified as a prehospital death by the HMCAS.

The patient data was classified by date of injury, age, sex, nationality, mechanism of injury [MOI], severity of injury, location of medical consultation, and clinical outcome.

WRI deaths were further classified and analyzed by date, timing of death [prehospital or in-hospital], MOI, location of injury, and cause of death.

Descriptive analyses were reported as frequencies and percentages for categorical variables. Continuous variables’ central tendency was described using means and medians. All statistical analyses were conducted using the SPSS 21.0 Statistical Package.

## Results

### Overall data management

**Figure 1.**
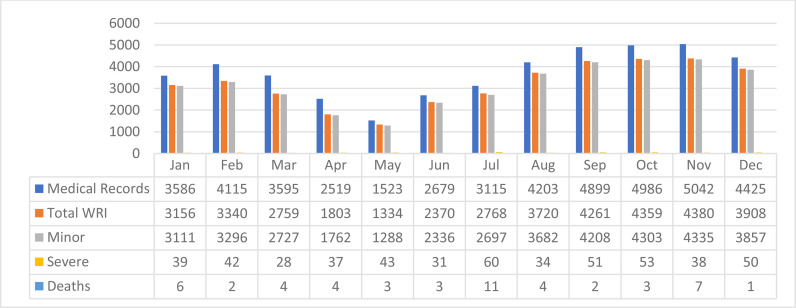
Work-Related Injuries and Medical Records, WURQ, January - December 2020.

During the 12-month study period, 44,687 medical records were reviewed. There was an overall 23.4% increase in the number of records reviewed and in the number of WRIs occurring every month, from January 2020 to December 2020 (Figure 1). The range of total monthly WRIs, 1803 to 4380 cases, is indicative of the effect of the COVID-19 restrictions and their lifting as well.^12^ The increasing trend is primarily from the increase in minor WRIs that started in June 2020, after the lowest number of minor WRIs in May 2020. The number of medical records reviewed reflects not only the number of WRIs but also the number of records that were duplicates or erroneously classified.

### WRI deaths

**Figure 2.**
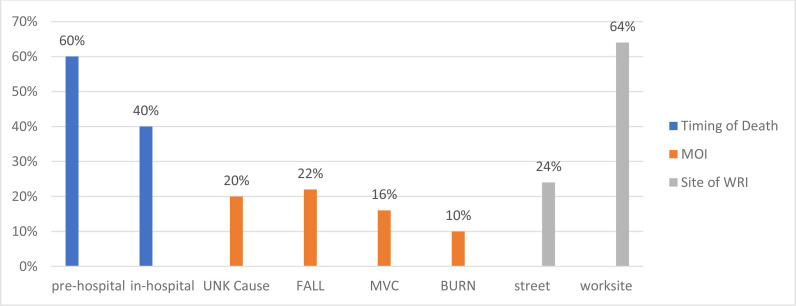
Key Characteristics of WRI Deaths, WURQ, January - December 2020UNK, unknown cause; MVC, motor vehicle crash; FALL, fall from height; BURN, thermal or chemical burns; MOI, mechanism of injury; WRI, work-related injury.

There were 50 deaths caused by work-related injuries out of a total worker population of 2,174,828 [2.29 occupational fatalities per 100,000 workers] in Qatar in 2020 [13], with a range from 1 to 11 and an average of 4.2 per month (Table 1)]. The majority of WRI deaths [30 out of 50] were in the prehospital setting [60%] with the majority of injuries [32] occurring at the worksite [64%] and 22% due to falls. Forty percent of WRI deaths [20 out of 50] occurred during the patient’s in-hospital stay. One-fifth of these deaths [10 out of 50] were due to “unknown” causes (Figure 2).

### Mild to severe WRIs

In Qatar in 2020, 506 workers sustained severe WRIs [23.26 severe occupational injuries per 100,000 workers], with a monthly range from 28 to 60 and an average of 42.2 per month, and 37,601 workers sustained mild to moderate WRIs [a WRI rate of 1728.91 mild to moderate occupational injuries per 100,000 workers], with the number of mild to moderate WRIs ranging from 1288 to 4335 and an average of 3113.4 WRIs per month.

### Nationality

**Figure 3.**
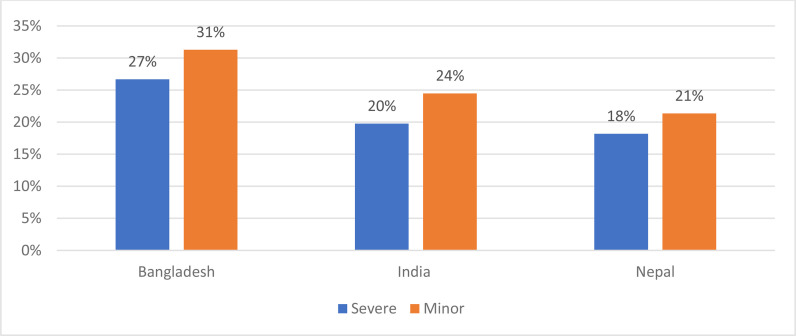
Mild and Severe WRIs by Nationality, WURQ, January - December 2020.

Two-thirds to three-fourths of severe and mild WRIs [65%–76%] affected workers from three countries [Bangladesh, India, and Nepal]. Workers from South Asia comprised a significant majority [78%–85%] of severe and mild WRI patients (Table 2 and Figure 3).

### MOI

**Figure 4.**
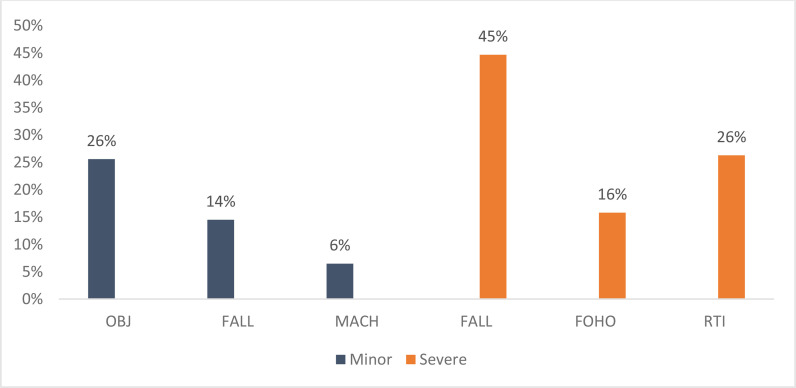
Mild and Severe WRIs by Mechanism of Injury, WURQ, January - December 2020OBJ, contact with an inanimate object; FALL, fall from height; MACH, machinery-related injury; FOHO, falling heavy object; RTI, road traffic-related injury.

There is a difference in the leading mechanisms of WRI when comparing them by the severity of injury. The severe WRIs are most commonly due to construction industry-related injuries, namely, falls [226 out of 506] from height [45%] and falling of heavy objects [FOHO] [80 out of 506] [16%]. It is noteworthy that road traffic injuries (RTI), to vehicle occupants/drivers, pedestrians, motorcycle drivers, and cyclists make up one-fourth [133 out of 506] of all severe WRIs. These three leading mechanisms of injury comprised at least 85% of all WRIs [439 out of 506] in Qatar in 2020 (Table 3 and Figure 4).

Mild WRIs are mostly due to excessive force from an inanimate object [OBJ], falls, and machinery-related injuries. The number of OBJ type injuries had the most dramatic increasing trend, increasing by 516% when comparing February [205] with November [1,263] 2020, as COVID-19 restrictions were lifted. Mild WRIs affecting motorcycle drivers were also noted to increase during the study period.

### Age group

There were minimal differences in the age groups most affected by severe and mild WRIs. The 25–34 years age group was slightly overrepresented [40% and 44%] as victims of WRIs, given that they only represent 37.3% of the total labor force in Qatar [13]. The second leading age group was proportionately represented among WRI victims [32% and 30%] as their age group made up 30.7% of the labor force in Qatar.^13^ (Table 4)

## Discussion

This study shows that, with an existent methodology and database, the task of collecting nationally representative data on mild, moderate, severe, and fatal WRIs in Qatar can be accomplished by a small team of dedicated data encoders, a database manager, a liaison and communications officer, with epidemiologic/statistical oversight. In 2020, there were 50 fatal WRIs, 506 severe WRIs, and 37,602 mild to moderate WRIs. On the average, there are 4.2 fatal WRIs [2.5 prehospital and 1.7 in-hospital deaths], 42.2 severe WRIs, and 3133.5 mild to moderate WRIs in Qatar every month. More than 95% of the WRIs affected male workers, and they are most accurately described as: 1) from five South Asian countries [Bangladesh, India, Nepal, Pakistan, and Sri Lanka]; 2) within the 25–34 years age group; 3) injured by impact from an inanimate object [OBJ]; and 4) while working as a general laborer at their worksite.

The data from this study was collected from almost all potential points of healthcare delivery for victims of WRI, making this the most recent and nationally representative description of the direct healthcare burden of mild, moderate, severe, and fatal WRIs in Qatar. The mechanism of identifying WRIs, applied by the WURQ registry, relied on an internationally accepted definition and a mandatory data field that had to be accomplished by the patient’s nurse or healthcare provider before a patient could be moved to another hospital location, admitted, or discharged. The grant-specific, simplified, and work-specific nature of the data collected as well as the prospective nature of the first 3 months of data collection minimized the “traditional” limitations that accompany retrospective data collection. Furthermore, each of the data elements was already being collected, prior to the study, and minimal training for new data entry or refresher courses were needed. This was the first local study that made use of prehospital data on work-related injuries, from the ambulance service, in Qatar. It is the first to accurately describe fatal WRIs that occur at the scene, a limitation reported by Tuma et al. in their description of work-related falls from height.^14^ With the addition of this data, a national WRI death rate, per 100,000 workers, can be reported for the first time. Due to the unique lockdowns and work activity restrictions mandated during the COVID-19 pandemic, the WRI data from 2020 may not be truly representative of the true epidemiology of WRIs in Qatar. If anything, this report, based on WRIs occurring during a year with changing COVID-19 restrictions for work activities in Qatar, is an undercount of the true health burden that WRIs would cause during a “regular” work year. The US reported a 10.7% decrease, and Singapore reported a 5.2% decrease in the total number of fatal WRIs in 2020.^15,16^ Unfortunately, we are unable to make a similar comparison because of the unavailability of data for WRIs in Qatar in 2019.

Historically, the male predominance among the victims of WRI in Qatar is a fact that has been reported previously.^17^ However, in communication with representatives from the Primary Health Care Corporation [PHCC], the authors were informed that female workers, primarily working as domestic helpers who suffer mild to moderate WRIs, might not seek medical consultation at any of this study’s data source locations. They report that they would rather be brought to PHCC clinics. As a result, this report may under report the mild to moderate WRIs suffered by this selected female worker population but, given that women do not work in high-risk industries or jobs, this number may not be a significant one. Efforts are being taken to apply the same mandatory field in the electronic medical records of the PHCC to address this potential gap in information collection.

The majority of the WRI deaths in this report occurred in the prehospital setting [60%] with their injury occurring at the worksite [64%]. While this proportion seems very high, it must be noted that this is much lower compared with pediatric injury deaths [93%] and those of all road traffic injury deaths [84%] in Qatar.^18,19^ This figure is almost identical to that reported for construction-related falls in Qatar [58.6%], by Tuma et al. in 2013.^14^ However, the major difference was that the previous report did not include data from the HMCAS. Given the identification of the construction industry as one of the highest risk occupations in the world,^10^ the previous report most likely provided an underestimate of the proportion of prehospital deaths from construction-related falls in Qatar during that period, 2007–2008. Regardless of the industry or occupation, this current report cannot overemphasize that a greater focus must be placed on the primary prevention of fatal WRIs, through proven interventions, such as the enforcement of mandatory personal protective equipment [PPE] use, worksite inspections, and incident investigations to further reduce the number of prehospital WRI fatalities.^14^

Forty percent of WRI deaths occurred during the patient’s in-hospital stay. All of these deaths occurred while the patients were under the care of the team at the HTC, the national trauma center of Qatar that is the hub of the internationally accredited national trauma system.^20^ All of these deaths underwent a thorough review of quality improvement and patient safety processes and outcomes as described elsewhere.^20^ The recommendations that arise from the results of the quality improvement evaluations of these cases must be used to complement WRI death reports. This essentially closes the loop and provides solid information for improving the secondary prevention of WRI deaths that occur during the patient’s hospital stay.

One-fifth of these deaths were due to “unknown” causes and, despite a thorough description of the nationalities of those with severe WRIs,^21^ many of their nationalities were undetermined. Since the majority of these “unknown” deaths occurred in the prehospital setting, this figure represents lack of a closure loop between incident investigators and the WURQ team. Additionally, the hectic prehospital environment is not an ideal setting to collect such data that even the identification of the patient may be delayed for 24–48 hours after admission. This calls attention to the need for deeper investigation of each WRI death and improvements in the linkage, accuracy, and quality of existent databases that capture WRI deaths.

Given the dearth of data on the breakdown by nationality, of Qatar’s labor force, it is difficult to say whether the leading nationalities represented in WURQ is a proportionate or a disproportionate representation of WRIs affecting workers from these countries. Historically, best evidence for nationality breakdowns of the resident population of Qatar have not been confined to workers only. Regardless, this data from WURQ provides a road map for future OHS educational, training, and communications programs. The potential for these countries’ embassies or their community and professional groups to serve as foci for outreach and injury prevention or worker safety programs must be fully explored and considered. The goal should be to provide culturally, linguistically, and epidemiologically focused worker safety education and awareness for those at the highest risk for WRIs. The Qatar office of the ILO, in conjunction with the MOL, has already begun this process.^22^

Fatal WRIs were most commonly caused by a fall from height [FALL] at a worksite that occurred in the prehospital setting. Overall, 0.13% of all WRIs recorded in WURQ resulted in a fatality, but this does not acknowledge the inherently nonfatal nature of the majority of the mild to moderate injuries. A more pragmatic mortality rate should take into account the in-hospital mortality rate of patients with severe injuries, 20 out of 506 severe WRIs or 3.9%. However, does either truly reflect the occupational health situation in any country? What is a globally accepted indicator that can be compared between countries and over time? One that will reflect the state of not only the medical response to WRIs but also the state of primary prevention for WRIs?

For the first time, a national occupational fatality rate based on WURQ and the 2020 Labour Force Survey can be reported for Qatar, which are 50 WRI deaths per 2,174,828 workers or 2.3 WRI deaths per 100,000 workers. This rate is 32% lower than the fatal occupational injury death rate in the US for 2020, 3.4 deaths per 100,000 workers,^15^ but still higher than the WRI fatality rate reported by Singapore, 0.9 deaths per 100,000 workers.^16^ The standardized occupational fatality rate in the EU was reported as 2.21 per 100,000 workers in 2018.^23^ Countries with more mature systems and institutions for occupational safety, such as Austria [4.3], France [3.7], Norway [3.1], Czechia [2.9], Spain [2.8], and Italy and Portugal [2.7], had higher occupational fatality rates in 2018 than Qatar in 2020.^24^ Of the countries that reported occupational fatality rates for 2020, the following reported rates are higher than Qatar: Turkey [5.95], Thailand [5.27], South Korea [4.65], Malta [2.67], and Estonia [2.32].^25^ This data allows a fair, objective, and globally accepted comparison of standard indicators of national occupational safety and renders claims in the popular press questionable and unsubstantiated.

Severe WRIs were most commonly caused by falls from height [45%] and FOHO [16%] at a worksite. These findings are consistent with previous reports^14,26^ and further emphasize the consistent need for increased worksite inspections in construction and worksites in Qatar. These efforts are being supplemented by ongoing training of more labor inspectors, culturally appropriate worker education, and awareness campaigns to increase the use of PPEs and address other potential risk factors for these injuries.^27^

One-fourth of severe WRIs were classified as work-related RTI. This is consistent with previous reports on work-related RTI,^28^ and some went further as to focus on worker pedestrians^29^ and heavy vehicles^30^ as areas of even more focused intervention for worker safety in Qatar. Clearly, in Qatar, WRIs and RTIs must be acknowledged for their multifactorial etiology but even more so for the need for multi-disciplinary collaboration, solutions, and interventions.

This initial annual report from the WURQ has set the baseline for the state of occupational safety in the country. WURQ has the potential to produce prospectively collected and real-time WRI data that could be used by worksite inspections and investigations teams, so they can conduct their work in a timely manner and make a positive impact on the safety of other workers who are still at that same worksite. The monthly reports can be used to monitor trends, evaluate programs, and identify new concerns for worker safety. The annual report can be used to compare and benchmark national occupational safety statistics against previous reports as well as against other countries.

A 1-month comparison of the WRI data submitted to the MOL as part of an annual report and the WURQ data was conducted for the first time. This comparison showed the level of incompleteness of each data source and their complementary nature. A major barrier to more efficient data comparison and was the patient confidentiality ruling on research grant data, set by the HMC Medical Research Center. This did not allow unique patient identifiers to be shared with other stakeholders.

The MOL has developed a focused safety training and messaging communications plan based on the data generated by WURQ for their worksite inspections teams, OHS safety officers, and general worker populations. These were created in various languages and pictographs, and plans are in place to disseminate them through multiple traditional and social media platforms. The 2020 WURQ data summary is serving as an evidence base for a communications plan that will address unsubstantiated claims about work-related deaths affecting workers in Qatar, ahead of the FIFA World Cup.^31^

Further efforts must be made to fully align the definitions and classifications of WRIs in WURQ, by mechanisms of injury, site of injury, industry/occupation, and diagnosis, with those of the other key stakeholders for OHS in Qatar, that is, MOL and MoPH. Consideration must be made for alignment with globally recognized and standard OHS benchmarks as well.

Repeated training for the healthcare personnel who accomplish the mandatory data linkage tool of WURQ must be performed. This is necessary given the constant recruitment of new staff and their rotation in other units. The plan is to incorporate this training in orientation of new staff and at regular, quarterly intervals for current staff, making use of online training modules that will be made mandatory for all staff.

Policies and agreements must be implemented to incorporate these additional data sources: PHCC clinics, e-Jaza database [where applications for sick leaves from work are filed electronically by government employees], private clinics/hospitals, military clinics/hospitals, and petrochemical company clinics.

In the absence of a suitable alternative source of data on WRIs in Qatar, it is recommended that WURQ be extended, sustained, and supported until an alternative database, which can provide similar or more extensive data on WRIs, is fully functioning.

The decision to sustain WURQ must take into account the following:

1. WURQ must be sustained as a working registry, no longer as a research grant or project. This is important as it will allow the sharing of data with unique patient identifiers, that is, the national identification number or Qatar ID. This will allow direct comparisons between OHS stakeholder’s databases and facilitate investigations of all WRIs and WRI deaths.
2. All efforts to ensure consistency of WRI data definitions, inclusion criteria, and classifications with national and global benchmarks must be prioritized.
3. Plans, timelines, and staff must be earmarked for training, re-training, and quality improvement of the data capture process for all future WURQ activities.
4. The estimates for manpower, equipment, and costs to support WURQ must consider the expected increase in the volume of WRIs as the local COVID-19 situation improves and restrictions are lifted.
5. Another factor to consider is that work activities will increase and accelerate as the FIFA World Cup 2022 start date comes closer. This will entail more time and staff to process the WRI data.
6. Estimates for support must also consider the need to incorporate newly identified data sources that have not yet been included in WURQ: PHCC clinics, e-Jaza database, private clinics/hospital, military clinics/hospitals, and petrochemical company clinics.
7. As the FIFA World Cup 2022 start date approaches, there may be a demand for empiric data on the state of worker health and safety in Qatar to counter misinformation being spread through mass media.

## Conclusions

In conclusion, collecting and reporting recent and representative national data on mild to fatal WRIs in Qatar is feasible. WURQ has described WRIs in Qatar using a purpose-built and nationally linked occupational injury registry. Occupational injury and injury fatality statistics, for Qatar in 2020, are lower than or comparable with those from other high-income countries. This data can be used to inform worksite inspections, investigations, worker safety education, environmental improvements, and injury prevention programs to make Qatar safer for all its workers. Until a more readily available or complete WRI database exists, every effort must be taken to ensure that WURQ is sustained and supported.

### Competing interests

The authors of this study do not have any competing interests

### Funding sources

The work has been funded by the following:

• Hamad Medical Research Centre
• International Labour Organization

### Author contributions

• Rafael Consunji
 o Study conception and design, data acquisition, analysis and interpretation
 o Preparing the manuscript
• Ayman El-Menyar
 o Study design
 o Revised and approved the final manuscript
• Nazia Hirani
 o Data acquisition and analysis
 o Revised and approved the final manuscript
• Aisha Abeid
 o Study conception and design, data acquisition, analysis and interpretation
 o Revised and approved the final manuscript
• Hassan Al-Thani
 o Study conception and data acquisition
 o Revised and approved the final manuscript
• Muhammad S Hardan
 o Data acquisition
 o Revised and approved the final manuscript
• Sailesh Chauhan
 o Data acquisition
 o Revised and approved the final manuscript
• Hasan Kasem
 o Data acquisition
 o Revised and approved the final manuscript
• Ruben Peralta
 o Study conception and design, data interpretation
 o Revised and approved the final manuscript

All authors have read and approved the final manuscript

Ethical approval for this study was obtained from the Research Ethics Committee, Medical Research Center, Hamad Medical Corporation, Doha, Qatar [MRC No: MRC-01-19-103 and MRC-01-19-103/ADHOC-01].

### Appendix A: Data Collection Pathway for WURQ 2 and WURQ 3

WURQ 2 data collection took place prospectively from February 1 to April 30, 2020. Patient records were reviewed to identify and remove duplicates; this data was further classified, organized, and analyzed in monthly reports. These reports were completed and submitted on the 10th of each succeeding month for these 3 months.

Data for all of the remaining months of 2020 for WURQ 3 was collected retrospectively from all the listed data sources for WRIs and WRI fatalities in Qatar. These records were reviewed to identify and remove duplicates; this data was further classified, organized, and analyzed in monthly reports.

### Appendix B:

The specific objectives of WURQ 2 are the following:

• W2-1.) To establish a “proof of concept” that the unified registry for WURQ is a viable and sustainable database and provide an estimate on workload, staffing, and managerial needs for the same, for future planning and possible institutionalization/adoption by an appropriate government ministry.
• W2-2.) To prospectively collect data, on WRIs, from all linked local data sources.
• W2-3.) To collate and analyze the data from #1, to accomplish the following:
 o To provide de-identified and aggregated WRI data to inform stakeholders in occupational safety in Qatar.
 o To estimate the costs and healthcare resources utilized by the care of WRIs in Qatar.
 o To utilize the data to inform the creation of locally accurate, focused and timely programs that will improve worker safety in Qatar.
 o To identify areas for future prioritization and focus for worker/occupational safety policies, research and programs in Qatar.

### Appendix C:

**Figure 5. fig5:**
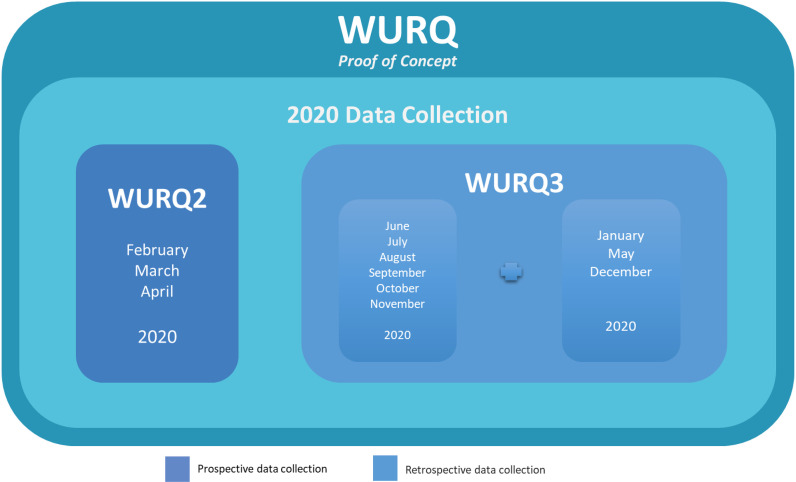
Data collection pathway for WURQ2 and WURQ3.

The specific objectives of WURQ 3 are the following:

• W3-1.) To use the WURQ data as the basis for both strategic and specific interventions by the OHS Unit of the Labour Inspection Department in MOL. This could include the following:
 o Planning of OHS inspections and communication campaigns based on the most complete, reliable data and assessment of their effectiveness, for example, measuring the impact of new legislation and practical guidance to mitigate the risk of heat stress in the summer.
 o Investigations of specific WRIs. A protocol will be adopted and tested for sharing of information on WRIs among relevant authorities to allow for follow-up action.
• W3-2.) To fine-tune the current WUR-Q system for collecting the most complete and accurate data on fatal and nonfatal WRIs. For example:
 o Data entry: Reporting systems may need to be adjusted, for example, to avoid duplications, and medical professionals trained to ensure more accurate data entry.
 o Incorporation of MOL data: Checking whether the information submitted to MOL by employers, MOI, or other is already captured by the HTC and QRCS data. This would support MOL’s efforts to improve reporting from employers.
 o Analysis: This could include modifying the categories of WRIs and the standard tabulations.
• W3-3) To learn from the WUR-Q experience to generate a set of concrete recommendations on the development of a sustainable model for the collection of timely and reliable data on fatal and nonfatal WRIs, in coordination with similar initiatives that have been developed at the national level, including within the inter-ministerial task force on safe and healthy employees.
• W3-4) To document the processes and outcomes of the research, or elements of the research, in analytical and academic reports, only where prior permission is granted from HMC MRC and MOL.[Fig fig5]

## Figures and Tables

**Table 1:** Key characteristics of WRI deaths [WURQ, January–December 2020].

	Jan	Feb	Mar	Apr	May	Jun	Jul	Aug	Sep	Oct	Nov	Dec	Total
Prehospital	6	1	4	3	0	3	10	1	0	1	0	1	30
In-hospital	0	1	0	1	3	0	1	3	2	2	7	0	20
UNK Cause	1	0	1	2	0	2	4	0	0	0	0	0	10
FALL	2	1	1	1	1	0	1	1	0	1	2	0	11
MVC	0	0	2	0	0	0	0	0	1	1	3	1	8
BURN	0	0	0	0	0	0	5	0	0	0	0	0	5
Street	1	0	1	1	0	0	1	1	1	2	3	1	12
Worksite	4	0	3	3	3	3	8	2	1	1	4	0	32

UNK, unknown cause; MVC, motor vehicle crash; FALL, fall from height; BURN, thermal or chemical burns.

**Table 2:** Mild and severe WRIs by nationality [WURQ, January-December 2020].

	Severe													
	Jan	Feb	Mar	Apr	May	Jun	Jul	Aug	Sep	Oct	Nov	Dec	Avg	% of total
Bangladesh	11	12	7	11	4	8	16	8	14	17	11	16	11.3	27%
India	5	6	5	6	10	5	19	5	9	10	8	312	8.3	20%
Nepal	8	15	8	6	4	5	10	7	9	8	7	5	7.7	18%
Egypt	3	2	0	3	6	2	4	4	2	3	5	2	3.0	7%
Pakistan	5	1	1	4	3	1	2	3	6	2	0	6	2.8	7%
Sri Lanka	1	0	1	3	5	1	4	1	4	5	3	0	2.3	6%
Mild													
	Jan	Feb	Mar	Apr	May	Jun	Jul	Aug	Sep	Oct	Nov	Dec	Avg	% of total
Bangladesh	885	979	797	513	339	685	812	1211	1432	1474	1393	1242	980.2	31%
India	767	833	700	414	299	572	720	836	1056	972	1062	968	766.6	24%
Nepal	683	711	567	374	277	493	571	813	871	928	922	821	669.3	21%
Sri Lanka	146	154	119	81	66	120	128	205	202	206	228	217	156.0	5%
Egypt	163	151	115	86	55	106	91	154	147	158	167	126	126.6	4%
Pakistan	112	121	119	77	59	93	111	138	147	143	155	104	114.9	4%

**Table 3:** Mild and severe WRIs by leading mechanisms of injury [WURQ, January–December 2020].

	Severe														
	Jan	Feb	Mar	Apr	May	Jun	Jul	Aug	Sep	Oct	Nov	Dec	Total	Avg.	% of total
FALL	14	19	12	17	19	13	35	16	20	26	17	18	226	18.83	45%
RTI	12	13	7	9	11	8	8	11	15	16	12	11	133	11.08	26%
FOHO	10	5	2	6	9	6	12	5	7	4	5	9	80	6.67	16%
	Mild														
	Jan	Feb	Mar	Apr	May	Jun	Jul	Aug	Sep	Oct	Nov	Dec	Total	Avg.	% of total
OBJ	952	205	582	342	378	583	640	1080	1279	1198	1263	1120	9622	801.83	26%
FALL	466	456	369	241	170	339	407	495	582	614	696	615	5450	454.17	14%
MACH	166	223	178	128	77	146	201	211	279	285	295	249	2438	203.17	6%

OBJ, contact with an inanimate object; FALL, fall from height; MACH, machinery-related injury; FOHO, falling heavy object; RTI, road traffic-related injury.

**Table 4:** Mild and severe WRIs by age group [WURQ, January–December 2020].

	Severe														
Age Group [years]	Jan	Feb	Mar	Apr	May	Jun	Jul	Aug	Sep	Oct	Nov	Dec	Total	Avg.	% of total
25–34	15	17	11	14	15	14	24	16	21	19	15	19	200	16.67	40%
35–44	13	17	9	9	16	4	19	11	21	16	9	18	162	13.50	32%
	Mild														
Age Group [years]	Jan	Feb	Mar	Apr	May	Jun	Jul	Aug	Sep	Oct	Nov	Dec	Total	Average	% of total
25–34	1400	1455	1162	771	567	1009	1189	1634	1850	1952	1997	1746	16732	1394.3	44%
35–44	860	872	833	519	362	730	798	1126	1294	1324	1259	1186	11163	930.3	30%
